# The G215R Mutation in the Cl^−^/H^+^-Antiporter ClC-7 Found in ADO II Osteopetrosis Does Not Abolish Function but Causes a Severe Trafficking Defect

**DOI:** 10.1371/journal.pone.0012585

**Published:** 2010-09-07

**Authors:** Patrick Schulz, Johannes Werner, Tobias Stauber, Kim Henriksen, Klaus Fendler

**Affiliations:** 1 Department of Biophysical Chemistry, Max Planck Institute of Biophysics, Frankfurt, Germany; 2 Leibniz-Institut für Molekulare Pharmakologie (FMP) and Max-Delbrück-Centrum für Molekulare Medizin (MDC), Berlin, Germany; 3 Nordic Biosciences, Herlev, Denmark; Indiana University, United States of America

## Abstract

**Background:**

ClC-7 is a ubiquitous transporter which is broadly expressed in mammalian tissues. It is implied in the pathogenesis of lysosomal storage disease and osteopetrosis. Because of its endosomal/lysosomal localization it is still poorly characterized.

**Methodology/Principal Findings:**

An electrophysiological characterization of rat ClC-7 using solid-supported membrane-based electrophysiology is presented. The measured currents show the characteristics of ClC-7 and confirm its function as a Cl^−^/H^+^-antiporter. We have used rat ClC-7 in CHO cells as a model system to investigate the functionality and cellular localization of the wt transporter and its variant G213R ClC-7 which is the analogue of human G215R ClC-7 responsible for autosomal dominant osteopetrosis type II. Our study shows that rat G213R ClC-7 is functional but has a localization defect in CHO cells which prevents it from being correctly targeted to the lysosomal membrane. The electrophysiological assay is tested as a tool for drug discovery. The assay is validated with a number of drug candidates. It is shown that ClC-7 is inhibited by DIDS, NPPB and NS5818 at micromolar concentrations.

**Conclusions/Significance:**

It is suggested that the scenario found in the CHO model system also applies to the human transporter and that mislocalization rather than impaired functionality of G215R ClC-7 is the primary cause of the related autosomal dominant osteopetrosis type II. Furthermore, the robust solid-supported membrane-based electrophysiological assay is proposed for rapid screening for potential ClC-7 inhibitors which are discussed for treatment of osteoporosis.

## Introduction

Members of the family of CLC chloride channels and transporters have received increasing attention in the last years because of their important physiological functions and their implication in pathogenesis. They are, therefore, important targets for drug discovery. CLC proteins can be divided into a group localized in the cellular plasma membrane and one localized in the membranes of intracellular organelles. Interestingly, the members of the first group are Cl^−^ channels (ClC-K, ClC-1 and ClC-2) while the members of the latter group are putative Cl^−^/H^+^-exchangers. Antiport activity was demonstrated for ClC-4 and 5 [1], [2], ClC-6 [3] and ClC-7 [4], [5]. Some of the CLC transporters in intracellular membranes are still poorly characterized because of a lack of sensitivity of conventional electrophysiological techniques for these transporters. Especially for ClC-7 attempts to bring this transporter to the plasma membrane for electrophysiological characterization have failed so far [6].

In this paper we therefore concentrate on ClC-7, a ubiquitous transporter which is broadly expressed in mammalian tissues [6]. It is found in lysosomes, late endosomes and in the ruffled membrane of osteoclasts [7], [8]. In lysosomes, ClC-7 represents the major Cl^−^ pathway [4], [9]. Recent findings indicate that the loss of ClC-7 does not alter lysosomal acidification [7], [10] but affects chloride accumulation [5]. In the ruffled membrane of osteoclasts, ClC-7 is essential for bone resorption [8]. The ruffled membrane lines the resorption lacuna, a specialized acidic compartment for degradation of the organic bone matrix. The resorption lacuna is acidified by a specialized V-type H^+^ ATPase [11], while ClC-7 might provide a charge compensation for effective acidification or be involved in the exocytic build-up of the ruffled border [8]. Indeed, ClC-7 knockout mice display severe lysosomal storage disease and an impaired bone resorption phenotype [8], [12].

For effective lysosomal function and bone resorption ClC-7 requires a β-subunit, Ostm1. Although ClC-7 is correctly targeted to lysosomes, association with Ostm1 enhances ClC-7 stability probably by protecting it from degradation [10].

Mutations in the *CLCN7* gene have been associated with pathological phenotypes such as different types of osteopetrosis [13], [14], [15], [16]. For example, the G215R mutation (of human ClC-7) is the most frequent cause of autosomal dominant osteopetrosis type II (ADO II) [16], [17]. In particular, ADO II osteoclasts have decreased acidification leading to reduced resorption of mineralized bone [18], [19]. The same authors have suggested that expression and localization of ClC-7 is normal in ADO II patients implying that this pathology is caused by a reduced functionality of G215R ClC-7.

In the following we present an electrophysiological characterization of rClC-7 using solid-supported membrane-based electrophysiology (SSM-based electrophysiology) [20]. Investigation of ClC-7 by standard electrophysiology was not possible due to the lack of ClC-7 expression in the plasma membrane. We investigated function and localization of G213R rClC-7 to decide whether mislocalization or impaired functionality of G215R ClC-7 is responsible for ADO II.

Furthermore, ClC-7 inhibition is discussed for treatment of osteoporosis [21], [22], [23], [24], [25]. However, a robust assay for rapid screening of drug candidates is still not available. We, therefore, tested SSM-based electrophysiology as a tool for drug discovery and validated it with a number of inhibitors.

## Materials and Methods

### Chemicals

A 1 mM octadecyl-mercaptan (C18-mercaptan, Aldrich, Steinheim, Germany) solution in ethanol was used for the incubation of the gold sensors. The lipid film forming solutions was diphytanoyl-phosphatidylcholin (Avanti Polar Lipids Inc. Pelham, AL) and octadecylamin (60∶1, wt/wt, 98%, Riedel-DeHaen AG, Seelze Hannover, Germany). Phloretin, Luteolin, Kaempferol, Tamoxifen, Genistein, Quercitin, Furosemide, 2-[3-(trifluoromethyl)anilino]nicotinic acid (NFA), 4,4′-diisothiocyanatostilbene-2,2′-disulfonic acid (DIDS), 5-nitro-2-(3-phenylpropylamino)benzoic acid (NPPB), p-chlorophenoxy-acetic acid (CPA), p-chlorophenoxy-propionic acid (CPP), indanyloxyacetic acid 94 (IAA-94) and 9-anthracene-carboxylic acid (9-AC) were obtained from Sigma-Aldrich. NS5818 and NS3736 were synthesized at Bioduro, (Beijing, China).

### Molecular Biology

Rat ClC-7 was cloned with a C-terminal GFP-fusionprotein into pcDNA5/FRT/TO using HindIII and EcoRV. Point mutations were introduced using the QuikChange Site-Directed Mutagenesis Kit (Stratagene, La Jolla, USA) at position 64 of GFP (Phe to Leu, primer: 5′ - CCA ACA CTT GTC ACT ACT CTC ACT TAT GGT GTT CAA TGC - 3′; 5′ - GCA TTG AAC ACC ATA AGT GAG AGT AGT GAC AAG TGT TGG - 3′) resulting in EGFP and at position 213 of rClC-7 (Gly to Arg, primer: 5′ - CAG ATC AAG TGC TTC CTC AAT AGG GTG AAG ATC CCC CAG GTG GTG - 3′; 5′ - CAC CAC GTG GGG GAT CTT CAC CCT ATT GAG GAA GCA CTT GAT CTG - 3′) resulting in the mutant comparable to the human G215R. Mouse Ostm1 was obtained in pFROG and could be transfected directly.

### Cell culture

The pcDNA5 constructs were co-transfected with pOG44 into a Flp-In™ CHO cell line (all Invitrogen™, Karlsruhe, Germany) using Effectene (Qiagen, Hilden, Germany). Stable transfectants were selected by maintaining the cells in HAM's F-12 medium (PAA Laboratories, Cölbe, Germany), containing 10% FCS, 1% Penicillin/Streptomycin and 600 µg/ml Hygromycin for 10–14 days. Medium was replaced every 2–3 days. These cell lines, constitutively and homogeneously expressing the ClC-7 fusion protein, were additionally transfected with pFROG-mOSTM1 and selected for stable transfectants with 500 µg/ml G418. For single clone isolation, cells were seeded at a theoretical concentration of one cell/well in 96well plates. After 7 days, when single colonies were visible, the clones were expanded and analyzed for their expression pattern using confocal microscopy. For membrane preparation, cells were grown in multiple 15 cm culture dishes. At a confluency of 90–95% cells were harvested and collected by centrifugation (800× g). Subsequently, pellets were flash frozen in liquid nitrogen and stored at −80°C until preparation.

### ClC-Ka expression

Human ClC-Ka (NM_004070) was stably co-expressed with its β-subunit barttin (NM_057176) in CHO cells using the bipromoter vector pBudCE 4.1 (Invitrogen). ClC-Ka was inserted via Hind III and Xba I downstream of the CMV promoter, while Barttin was inserted after the EF-1a promoter using Kpn I and Xho I. Stable clones were selected by 150 µg/ml zeocin (Invitrogen). Cells were cultured and harvested as described before.

### EcClC-1 expression

EcClC-1 was expressed in *E. coli* (BL21 DE3), purified as described before [26] and reconstituted into liposomes (*E. coli* polar lipids, LPR = 10) [27].

### Membrane preparation

Cell pellets were resuspended in buffer (10 mM TRIS, pH 7.5, 250 mM sucrose, 2 mM dithiothreitol (DTT) and 1 tablet Roche Complete™) and disrupted in a Parr Bomb (Parr Instruments Deutschland GmbH, Frankfurt/Main, Germany). Total membranes were collected by ultracentrifugation (30 min, 100.000× g @ 4°C) and fractionated by density gradient centrifugation (3 h, 100.000× g @ 4°C) [20] using a discontinuous sucrose gradient of 9%, 31%, 45% and 51%. Membrane bands at the gradient interfaces were collected, diluted 10 fold in resting buffer and pelleted by ultracentrifugation (30 min, 100.000× g @ 4°C). Protein concentration was determined by Bradford assay (BioRad, München, Germany). For long term storage, membranes were flash frozen in resting buffer (with 10% glycerol, 2 mM DTT and protease inhibitors) and stored at −80°C.

### Confocal Microscopy

To investigate the vesicular localization of the wild type (wt) and mutant ClC-7-EGFP fusion proteins, cells were grown on coverslips for 24 to 48 hours. After reaching a confluence of 50–80% cells were incubated with 50 nM LysoTracker®-Red for 15 min or 1 µM ER-Tracker™-Red for 30 min, respectively (Invitrogen). Samples were analyzed instantaneously with a confocal laser-scanning microscope (LSM 510, Zeiss) using the argon laser line at 488 nm for EGFP and a 543 nm HeNe laser for Lyso- and ER-Tracker excitation. Fluorescence was detected through a 500–550 nm band pass filter and a 560 nm long pass filter, respectively. Images were recorded with an Achroplan 63×/0.95 W water immersion objective in multi track mode with sequential excitation of EGFP and Tracker dye, respectively. LysoTracker®-Red was prevented from exposure to broadband illumination before confocal imaging to prevent photoconversion [28].

### Fluorimetric analysis of membrane fractions

For a quantitative analysis of the membrane fractions the EGFP fluorescence of the ClC-7 fusion proteins was analyzed using a microplate fluorescence reader (Lambda Fluoro, BIO-TEK Instruments, Winooski, USA). Membrane fractions were diluted to an equal concentration of 1 mg/ml in 50 µl buffer (60 mM HEPES, pH 7.2, 3 mM CaCl_2_, 300 mM NaAsp). EGFP was excited using a 480/20 nm band pass filter, fluorescence emission was detected through a 530/25 nm band pass filter. The samples were analyzed in Greiner 96well flat bottom plates, the optical position was the well bottom.

### Western blot

Expression of the ClC-7-EGFP fusion protein and vesicular distribution of the membrane fractions was tested by Western blot. 15 µg total protein were loaded on a 4–12% bis-tris gel. After electrophoretic separation, proteins were blotted on a nitrocellulose or PVDF membrane (Invitrogen) and blocked for 1 hour in 5% nonfat dry milk (PBS-T). The blot was incubated with a rabbit-anti-GFP antibody (Clontech, Mountain View, CA, USA) (1∶100) overnight at 4°C. Lysosome or ER-enriched membrane fractions were identified, using a monoclonal mouse anti-LAMP-1 (Abcam) and a polyclonal rabbit anti-calnexin antibody (Stressgen, Ann Arbor, MI, USA). For detection of mOstm1 and ClC-3 rabbit anti-mOstm1 [10] and anti-ClC-3 [29] antibodies were used. For detection, appropriate AP (alkaline phospatase) or HRP (horse radish peroxidase) coupled secondary antibodys (BioRad, München, Germany) were used. Finally, AP blots were developed using AP developer kit (BioRad, München, Germany). HRP blots were detected with SuperSignal® West Pico or Femto (Pierce, Epsom, UK) and developed on film (BioMax, Kodak, Stuttgart) using a CURIX 60 developer (AGFA, Köln). Densitometry analysis of Western blot bands was performed using ImageJ (NIH, Bethesda, Maryland, USA) [30].

### SSM-based electrophysiology

Electrophysiological measurements were performed on a commercial SURFE^2^R One instrument (IonGate Biosciences, Frankfurt, Germany). The experiments were carried out at room temperature (22°C). After the formation of the SSM [20], liposomes, membrane vesicles or membrane fragments (set to equal protein concentrations: ∼2 mg/ml) were adsorbed to the SURFE^2^R sensors (SSM-area ∼7 mm^2^) using a centrifugation procedure of 800× g for 45 min. For the ClC-7 measurements the 31/45% membrane fractions were used. Before mounting the sensors into the SURFE^2^R One instrument they were kept in Cl^−^ free buffer (60 mM HEPES, pH 7.2, 3 mM Ca-gluconate, 300 mM Na-aspartate). The solution exchange protocol consisted of 3 phases with a duration of 2 s each and a flow rate of 250 µl/s: a flow of nonactivating solution (NA) followed by activating solution (A) followed by nonactivating solution (NA). Solution A contained one or both transported substrates of ClC-7 (Cl^−^, H^+^). In the case of Cl^−^ concentration jumps, NA contained aspartate^−^ at the same concentration as Cl^−^ to minimize solution exchange artifacts. In addition all solutions contained a basic buffer which was 60 mM HEPES, pH 7.2, 3 mM CaCl_2_, 300 mM Na-aspartate at neutral pH. Detailed buffer compositions are given in the figure legends.

Different buffers or inhibitor concentrations could be applied automatically using the autosampler of the SURFE^2^R One instrument. After changing buffer conditions (eg. pH) the sensors were incubated for 10–15 minutes before measurement to allow equilibration of the internal vesicle volume to the buffer conditions.

## Results

**Figure 1:**
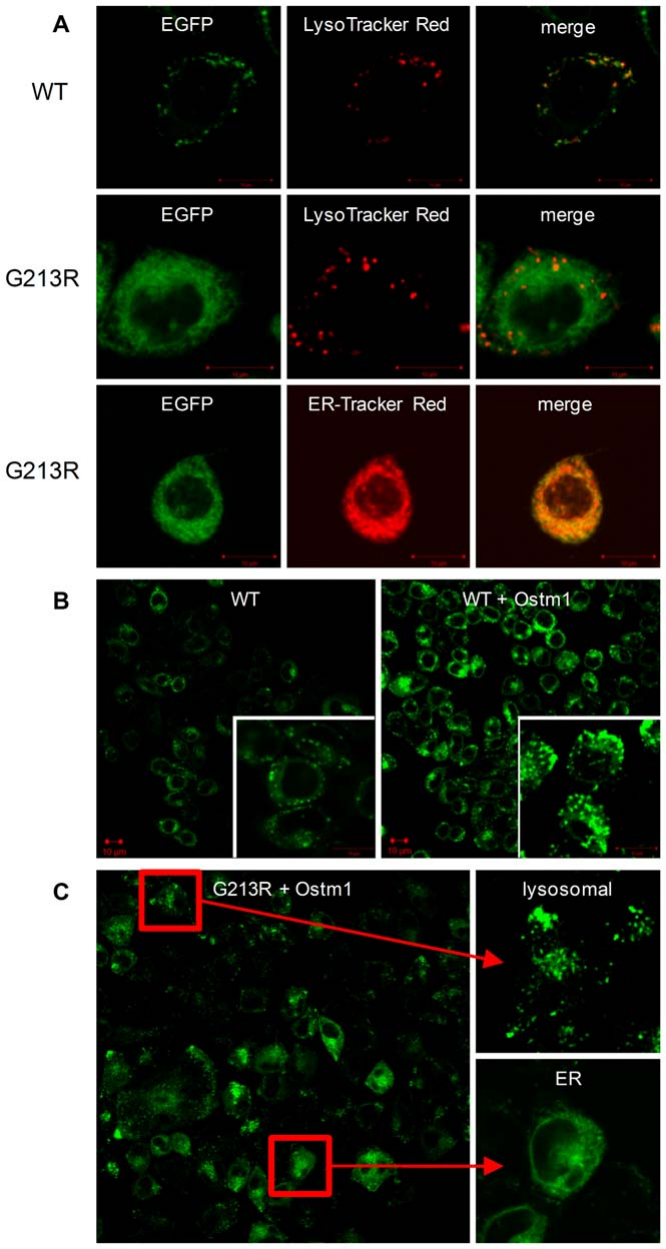
Localization of wild type and G213R ClC-7 in CHO cells. **A:** Confocal images of wt ClC-7-EGFP expressing living CHO cells stained with LysoTracker®-Red (top row). Images of EGFP fluorescence, LysoTracker®-Red fluorescence and the merge of both are shown. G213R ClC-7-EGFP stained with LysoTracker®-Red (middle row) and ER-Tracker™-Red (lower row). Individual fluorescence images and merge are shown. **B:** Co-expression of wt ClC-7-EGFP with its ß-subunit Ostm1. Compared images were recorded with fixed hardware settings and at constant experimental conditions. Localization of ClC-7 in Ostm1 expressing cells was not affected but a significantly increased EGFP fluorescence could be detected. **C:** Co-expression of G213R ClC-7-EGFP with Ostm1 partly (∼50%) restored lysosomal localization.

Wild type (wt) rat ClC-7 and its G213R variant (the rat homolog of human G215R) were cloned as fusion proteins with EGFP. The Flp-In™ expression system was used to generate stably expressing recombinant CHO-cells. Subsequently, the ClC-7 β-subunit mOstm1 [10], was co-expressed stably in these cell lines. Expression and localization were verified using confocal imaging. While the wt protein was expressed in small vesicular organelles which nicely co-localized with LysoTracker®-Red indicating a lysosomal localization (Fig. 1 A, top row), the G213R variant was broadly distributed inside the cells and did not co-localize with LysoTracker®-Red (Fig. 1 A, middle row). Instead, it co-localized with ER-Tracker™-Red indicating retention in the ER (Fig. 1 A, bottom row).

When the ß-subunit Ostm1 was co-expressed with wt ClC-7, the EGFP fluorescence and therefore the expression level of ClC-7 increased significantly but localization was not affected in all 12 analyzed clones (Fig. 1 B). In contrast, co-expression of Ostm1 with the G213R mutant altered the ClC-7 localization significantly. While the mutant alone showed homogeneous expression in the ER, the expression pattern became inhomogeneous after co-expression with Ostm1. In about 30–50% of the cells ClC-7 could be identified “re”-localized in lysosomes while in the rest still ER-localized expression was observed (Fig. 1 C). In addition, some cells could be identified where ClC-7 G213R was partly localized in both compartments, ER and lysosomes. To exclude variations in the expression level of Ostm1 to be responsible for this inhomogeneous distribution, 24 single clones of the stably expressing cell line were analyzed. The described expression pattern could be observed in almost all of the clones. No clone with complete lysosomal localization could be observed.

In all cell lines, expression in the plasma membrane could not be detected. Furthermore, control patch clamp experiments performed with those cells did not show significant chloride currents. The absence of ClC-7 expression in the plasma membrane of recombinant cell lines is in good agreement with the findings of previous studies [6], [7], [8], [10], [31]. However, plasma membrane expression of ClC-7 in HEK-cells was reported by Kajiya and coworkers [32].

**Figure 2:**
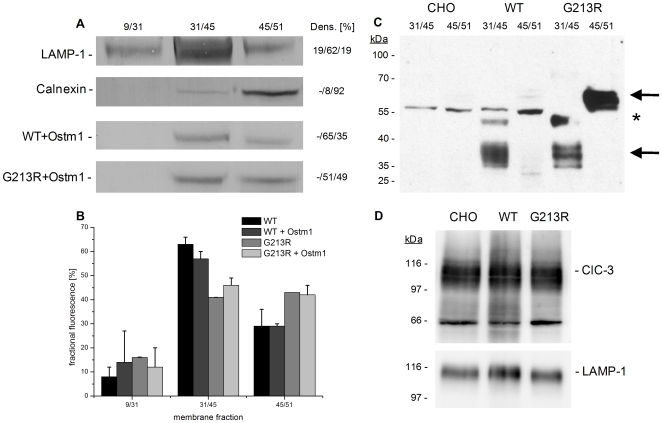
Activity and fractional distribution of wild type and G213R ClC-7. **A:** Western Blot of membrane fractions against GFP (ClC-7), LAMP-1 (lysosomes) and Calnexin (ER), showing lysosomes enriched in the 31/45% fraction and ER in the 45/51% fraction. Densitometric analysis of Western blot bands (Dens.): numbers give relative intergral band densities in % left to right lane. **B:** Quantitative fluorescence analysis of membrane fractions. Fractions were adjusted to same total protein concentration. Fractional fluorescence was normalized to the total fluorescence in all samples. Mean and standard deviation of 2–3 individual preparations. Wt ClC-7 is significantly enriched in 31/45%, while G213R is more equally distributed between 31/45% and 45/51%. Ostm1 alters G213R localization only slightly. **C:** Western Blot against Ostm1. Fraction 31/45% and 45/51% of CHO control cells (CHO), wt ClC-7+Ostm1 (WT) and G213R+Ostm1 (G213R). Arrows: full length Ostm1 (∼70 kDa) and cleaved fragment (∼35 kDa), asterisk indicating an unspecific band recognized by the Ostm1 antibody [10]. **D:** Western Blot against ClC-3 and LAMP-1 in fraction 31/45% of control CHO, wt ClC-7+Ostm1 and G213R ClC-7+Ostm1.

Due to the lack of ClC-7 expression in the plasma membrane, standard electrophysiology was not an option. Therefore, SSM-based electrophysiology was applied using membranes prepared from CHO cells. In an attempt to separate plasma membrane and internal membranes, sugar gradient fractionation was used as described in materials and methods. Western blot analysis of the different fractions revealed the following results (Fig. 2A): lysosomes (marker: LAMP-1) were found enriched in fraction 31/45%, while ER (marker: Calnexin) was dominantly present in 45/51%.

The ClC-7-EGFP fusion proteins (∼120 kDa) could be detected by Western blot in the fractions 31/45% and 45/51% (Fig. 2A). While the wt protein (co-expressed with Ostm1) was enriched in the lysosomal fraction 31/45% (Densitometric analysis revealed a relative integral band density of 65%), with only small amounts in the ER fraction 45/51% (Densitometry: 35%), the G213R mutant was more equally distributed between these fractions (Densitometry: 51 and 49%) showing a higher presence than wt in the ER fraction 45/51%.

To quantify the distribution more thoroughly we performed a fluorimetric analysis of the membrane fractions (Fig. 2B) taking advantage of the EGFP marker. Wt ClC-7 could be detected significantly enriched in the lysosomal fraction 31/45% as already demonstrated qualitatively in western blot analysis. Ostm1 co-expression did not significantly alter the distribution. G213R ClC-7 was detected almost equally distributed between the 31/45% and 45/51% fractions. Co-expression of Ostm1 slightly changed the distribution towards 31/45%.

Also the expression behavior of Ostm1 analyzed by western blot (Fig. 2C) is interesting. In wt ClC-7 cells the cleaved 35 kDa fragment of Ostm1 [10] could be dominantly detected in the lysosomal fraction 31/45%, while only small amounts of full length protein (∼70 kDa) were found in the ER fraction 45/51%. In G213R ClC-7 cells both bands could be detected, with a stronger population of full length protein in the ER fraction.

Because ClC-3 and ClC-6 have been shown to be shifted partially into lysosomal fractions upon lack of ClC-7 [9], we tested our lysosomal fractions (31/45%) for altered expression levels of other intracellular CLCs upon expression of the different CLC-7 forms. Only ClC-3 could be detected by western blot analysis in comparable amounts in control CHO, wt ClC-7+Ostm1 and G213R ClC-7+Ostm1 membranes (Fig. 2D), indicating that ClC-3 is not responsible for the altered transport activities found in the different preparations (see below).

**Figure 3:**
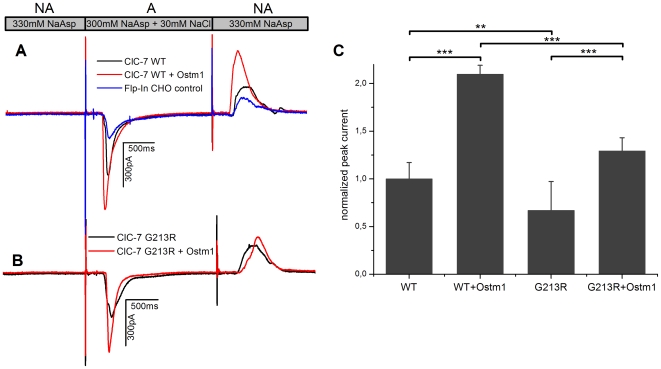
Transport activity of ClC-7 using SSM-based electrophysiology. **A:** Lysosomal fraction 31/45% of wt ClC-7 was adsorbed to the SSM-sensors and transport was initiated by a rapid concentration jump of 30 mM NaCl. Figure shows representative current traces of repetitive experiments. Flow protocol: nonactivating (NA) – activating (A) – nonactivating (NA). Solutions: (NA) 330 mM Na-aspartate (NaAsp), 3 mM Ca-gluconate, 60 mM HEPES pH 7.2, (A) 30 mM NaCl, 300 mM Na-aspartate, 3 mM Ca-gluconate, 60 mM HEPES pH 7.2. **B:** Transport activity of G213R ClC-7 using the same protocol as described above. **C:** Averaged signal amplitudes of all preparations, normalized to individual fractional EGFP fluorescence. Of each sensor at least 20 signals were recorded, 4–6 individual sensors were investigated for each preparation. Mean and standard deviation of 4–6 sensors each. For comparison, the corresponding non-normalized current amplitudes are given in the text. Student's *t*-test: * = p<0.05, ** = p<0.01, *** = p<0.001.

To determine ClC-7 protein activity, equal amounts of fraction 31/45% adjusted to the same total protein concentration were adsorbed to the SSM sensors (see materials and methods) and transient currents were generated by applying a fast concentration jump of 30 mM NaCl (Fig. 3). The peak current was used to quantify the transport activity of ClC-7 [20]. In this configuration negative currents are indicating the translocation of negatively charged ions (e.g. Cl^−^) towards the SSM. In view of the very gentle method of cell disruption by pressure homogenization [33], [34], lysosomes should still be intact after membrane preparation. However, only little is known about the adsorption geometry of membrane samples on the SSM.

While non-expressing Flp-In™ CHO cells yielded only very small endogenous currents (<150 pA, see Fig. 3A control), membranes of wt ClC-7 generated currents of 455±78 pA (6 sensors). When Ostm1 was co-expressed with wt ClC-7, currents increased significantly to 787±74 pA (6 sensors). Also G213R ClC-7 membranes yielded detectable currents (292±88 pA, 4 sensors), which increased (518±72 pA, 5 sensors) when Ostm1 was co-expressed. For a direct comparison of the transporter activity, the currents obtained with the different preparations were normalized to the corresponding EGFP fluorescence (Fig. 3C). Also in the normalized currents the Ostm1 induced increase of ClC-7 activity was observed in wt and G213R preparations while the G213R currents were slightly decreased (∼30%) compared to the wt measurements.

**Figure 4:**
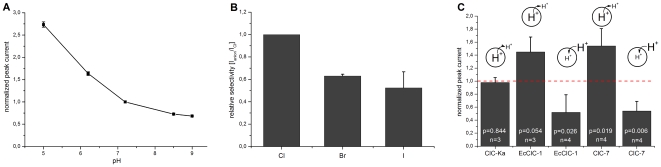
Electrophysiological characterization of ClC-7. If not otherwise indicated conditions as in Fig. 3. **A:** pH dependence of ClC-7 transport activity. Transport was initiated by a rapid concentration jump of 30 mM NaCl. Solutions: (NA) 330 mM Na-aspartate, 3 mM Ca-gluconate, 60 mM HEPES, (A) 30 mM NaCl, 300 mM Na-aspartate, 3 mM Ca-gluconate, 60 mM HEPES. HEPES buffer was replaced by MES or TRIS for acidic and basic conditions, respectively. After buffer exchange to a different pH, sensors were incubated for 15 min to allow equilibration to new conditions. **B:** Specificity of ClC-7 anion transport with Cl^−^>Br^−^>I^−^. Transport was initiated by a rapid pH jump (establishing a proton gradient pH 5.0 inside, pH 7.2 outside of the vesicle) with different anions present in all solutions. Solutions: (NA) 30 mM NaCl (NaBr, NaI), 300 mM Na-aspartate, 3 mM Ca-gluconate, 30 mM MES, pH 5.0. (A) 30 mM NaCl (NaBr, NaI), 300 mM Na-aspartate, 3 mM Ca-gluconate, 30 mM MOPS, pH 7.2). pH artifacts (same protocol, but substrate substituted by 30 mM Na-aspartate in A and NA, amplitude: ∼1 nA) were subtracted and the resulting currents were normalized to currents in the presence of NaCl. **C:** H^+^-coupled transporter activity of ClC-7 compared to other CLC proteins (ClC-Ka+barttin and EcClC-1). Transport was initiated by a simultaneous NaCl concentration (30 mM) and pH (ΔpH∼2) jump. Solutions pH 5.0_inside_–7.2_outside_: (NA) 30 mM Na-aspartate, 30 mM MES, pH 5.0, (A) 30 mM NaCl, 30 mM MOPS, pH 7.2. Solutions pH 9.0_inside_–7.2_outside_: (NA) 30 mM Na-aspartate, 30 mM TRIS, pH 9.0, (A) 30 mM NaCl, 30 mM MOPS, pH 7.2. In addition all buffers contained 300 mM Na-aspartate and 3 mM Ca-gluconate. pH artifacts (same protocol, substrate in A substituted by 30 mM Na-aspartate, amplitude: ∼1 nA) were subtracted and resulting currents were normalized to NaCl initiated currents in the absence of a pH gradient (pH 7.2). P = Student's *t*-test, significance level = 0.05.

In the following, the lysosomal 31/45% membrane fraction of wt ClC-7-Ostm1 expressing cells was used for further electrophysiological characterization of ClC-7. For the pH dependence, transient currents were initiated with a 30 mM NaCl concentration jump at different pH. Before measurement, sensors were incubated for 15 min in resting buffer at the desired pH to prevent pH gradients across the membranes of the adsorbed vesicles. Currents significantly increased at acidic pH with no saturation until pH 5.0 (Fig. 4 A).

Ion selectivity was determined for Cl^−^, Br^−^ and I^−^ (Fig. 4 B). Concentration jumps of Br^−^ and I^−^ generate large solution exchange artifacts due to the strong interaction of the anions with the lipid headgroups on the SSM [35]. Therefore, transport was initiated in this case by a proton concentration jump generating a pH gradient (pH 5.0 inside, pH 7.2 outside of the vesicle) in the presence of the different anions (30 mM Cl^−^, Br^−^ or I^−^). For comparison, the same proton concentration jump was applied in the absence of Cl^−^ and the resulting peak current (∼1 nA) subtracted. As displayed, ClC-7 is only weakly selective but shows a significant preference for Cl^−^ over Br^−^ over I^−^.

In addition to the determination of the ion selectivity, the experiment in Fig. 4B confirms H^+^-driven anion-transport which was further examined. Therefore, a 30 mM Cl^−^ concentration jump was applied together with inward directed and outward directed proton gradients and the signals were compared to that obtained in the absence of proton gradients (pH 7.2). In more detail: for the favorable pH gradient a combined Cl^−^ and proton concentration jump was applied (pH 5.0, no Cl^−^ inside and pH 7.2, 30 mM Cl^−^ outside the vesicle). The same proton concentration jump was applied in the absence of Cl^−^ (substituted by aspartate, amplitude: ∼1 nA) and the resulting peak current subtracted. The resulting difference signal was normalized to a simple Cl^−^ concentration jump (pH 7.2, no Cl^−^ inside and pH 7.2, 30 mM Cl^−^ outside the vesicle). For the unfavorable pH gradient the same procedure was used with pH 9.0 pH inside the vesicles. For comparison, this was done for different members of the CLC protein family: the human Cl^−^ channel ClC-Ka (+barttin), the well characterized Cl^−^/H^+^-antiporter EcClC-1 from *E. coli* and ClC-7 (Fig. 4C). While pH gradients had no effect on ClC-Ka (Cl^−^ channel) activity, EcClC-1 (Cl^−^/H^+^-antiporter) and ClC-7 currents were significantly modulated, depending on the direction of the gradient (Fig. 4C). Applying the gradient in the putative transport direction (lower pH inside, higher pH outside the vesicles) significantly increased (50%) the currents induced by a fast concentration jump of 30 mM NaCl. Applying the gradient against the transport direction (higher pH inside, lower pH outside the vesicles) decreased the currents by 40%.

**Figure 5:**
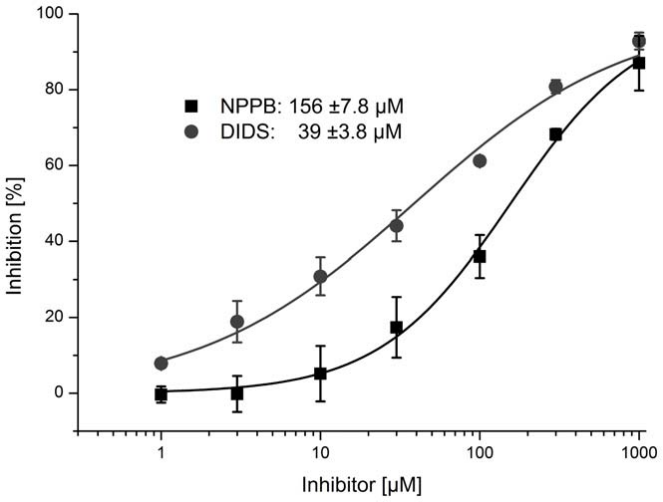
Inhibition of ClC-7. Several putative Cl^−^ channel inhibitors were tested for inhibition potency (Tab.1). ClC-7 was activated with 30 mM Cl^−^ concentration jump while different concentrations of inhibitors (1–1000 µM) were present in all buffers. The plots show mean inhibition values and standard deviation of at least 3 independent experiments (5 measurements per concentration each). The data was fitted with a Hill function, yielding IC_50_ values of 39±3.8 µM (Hill coefficient n = 0.6) for DIDS and 156±7.8 µM (Hill coefficient n = 1.0) for NPPB.

Specificity of the SSM-based electrophysiological ClC-7 assay was tested by screening a number of potential chloride channel inhibitors. Using the SURFE^2^R One instrument a semi- automatic screening procedure was established. 16 putative chloride channel inhibitors were characterized (Table 1) and IC_50_ values determined if possible. For that purpose, ClC-7 was activated by concentrations jumps of 30 mM NaCl in the presence of varying concentrations of inhibitors in all buffers. Before measurement, sensors were incubated for 5 min in chloride free buffer, containing the appropriate inhibitor concentration. IAA-94 and NS3736 generated large solution exchange artifacts probably due to strong interaction with the membranes and could, therefore, not be characterized. Furthermore Quercetin, Genistein, Luteolin and Phloretin precipitated at high concentrations (>100 µM), preventing the determination of a complete IC_50_. Quercetin and Genistein showed weakly inhibiting effects at 100 µM while CCP, CPA, 9-AC, Tamoxifen and Kaempferol did not inhibit ClC-7 currents in the investigated concentration range (<300 µM). Robust IC_50_ values could be determined for DIDS, NS5818 and NPPB. DIDS inhibited the currents with an IC_50_ of 39±3.8 µM, NS5818 with 52±8.0 µM and NPPB with 156±7.8 µM. As an example, the inhibition graphs for DIDS and NPPB are shown in Fig. 5.

**Table 1:** Analyzed Cl^−^ Channel inhibitors.

	Inhibitor	Range (µM)	Max (%)	IC_50_ (µM)
1	DIDS	1–1000	92	39±3.8
2	NS5818	1–300	70	52±8.0
3	NPPB	1–1000	90	156±7.8
4	Furosemide	3–1000	50	650
5	NFA	1–1000	30	-
6	Quercitin	1–300 #	25	-
7	Genistein	1–300 #	20	-
8	CCP	1–300	0	-
9	CPA	1–300	0	-
10	9-AC	1–300	0	-
11	Tamoxifen	1–300	0	-
12	Kaempferol	1–300	0	-
13	Luteolin	1–300 #	0	-
14	Phloretin	1–300 #	0	-
15	IAA-94	1–300	-	*
16	NS3736	1–300	-	*

16 different putative Cl^−^ channel inhibitors were tested. Concentration range (µM), maximum inhibition (%) and IC_50_ value (if determinable) is shown. *Could not be investigated (see text), #Precipitates at high concentrations (>100 µM).

## Discussion

### Solid-supported membrane-based electrophysiology allows characterization of ClC-7 expressed in CHO cells and confirms its function as a Cl^−^/H^+^-exchanger

This is the first electrophysiological characterization of ClC-7 in its native environment, i.e. in the lysosomal membrane. Transient currents observed with membrane preparations of CHO cells expressing rat ClC-7 show characteristics of ClC-7 transport [4], [5]: (1) strong pH dependence with an increased activity at acidic pH, (2) ion selectivity Cl^−^>Br^−^>I^−^ and (3) pH-gradient driven anion transport. In addition, unspecific Cl^−^ transport inhibitors like DIDS and NS5818 inhibited the currents with relatively high affinity (Table 1).

An essential feature of ClC-7 is its function as a Cl^−^/H^+^-exchanger [4], [5]. Coupling of ClC-7 Cl^−^ transport activity to a proton motive force is demonstrated by two independent experiments: (1) Anion specific currents driven by a proton gradient only (Fig. 4B). (2) Modulation of anion specific currents by a co- and countergradient of protons (Fig. 4C). The latter is a distinct property of a proton coupled exchanger and is also found in the related EcClC-1 transporter and not in the Cl^−^ channel ClC-Ka (Fig. 4C).

Note that pH regulatory effects cannot be excluded in this experimental approach. It is known that ClC-Ka currents in two-electrode voltage clamp measurements [36] are strongly dependent on extracellular pH. However, a pH dependence of the ClC-Ka currents was not detected in our experiments. This may be related to the design of our measurements where the external pH of the membrane vesicles was kept constant at pH 7.2, while the internal pH was altered (implying right side out oriented membrane vesicles).

SSM based electrophysiology was essential in this study, because ClC-7 is expressed in intracellular membranes i.e. late endosomes and lysosomes, which are not accessible for conventional electrophysiology. Unlike other intracellular transporters of the CLC family such as ClC-4 and ClC-5, which could be investigated in oocytes or mammalian cell lines because of a “spill-over” to the plasma membrane at high overexpression [24], [37], ClC-7 related currents could not be detected using whole cell patch clamp or two-electrode voltage clamp measurements by different authors in a variety of cell systems [6], [7], [8], [10], [31]. This agrees with our localization studies which show no expression in the plasma membrane within the limits of detectability. There are, however, reports of trans-plasma membrane currents in oocytes, osteoclasts and HEK cells that have been assigned to ClC-7 [32], [38]. The reasons for this discrepancy are still unclear.

### In CHO cells wild type ClC-7 resides in lysosomes while G213R ClC-7 is retained in the endoplasmatic reticulum

Fluorescence of the ClC-7-EGFP fusion protein was used to check the localization of ClC-7 expressed in CHO cells. In vivo ClC-7 resides in late endosomes, lysosomes and the ruffled membrane of osteoclasts [7], [8]. It is therefore not surprising to find wt ClC-7 in CHO cells predominantly in lysosomal membranes (Fig. 1A). In contrast, G213R ClC-7 shows a different intracellular distribution suggesting localization in the ER. This is quantitatively confirmed by densitometric analysis of ClC-7 western blots and by fluorimetric investigation of the different membrane fractions from sugar gradient fractionation. Wt ClC-7 is preferentially found in the 31/45% fraction which is enriched in lysosomal membranes while G213R ClC-7 is equally distributed between the lysosomal 31/45% fraction and the ER fraction 45/51% (Fig. 2B). Note, that densiometric and fluorimetric results are in good agreement. Therefore we propose that in CHO cells the G213R mutation is recognized by the ER-quality control, which prevents ClC-7 from leaving the ER where it is primarily formed and being transported to the lysosomal membrane.

Interestingly, Ostm1 significantly changes the expression pattern of G213R ClC-7, restoring the lysosomal localization to some extent. In addition, G213R ClC-7 co-expressing Ostm1 is characterized by a small preference for the lysosomal membrane fraction 31/45% used for the electrophysiological experiments (Fig. 2 B), resulting in an increase of the measurable currents. On the level of individual cell clones, an inhomogeneous pattern is found. Some cells show predominantly lysosomal, some cells predominantly ER localization. But Ostm1 significantly increases the lysosomal fraction (Fig. 1C). Because ClC-7 is not dependent on Ostm1 to reach the lysosomes in general [10], the function of Ostm1 in our experiments might be to compensate a putative folding defect caused by the G213R mutation. Thereby the protein it is no longer recognized by the ER quality control, resulting in a lysosomal “relocalization”. Previous studies indicated that Ostm1 is cleaved in or on its way to lysosomes [10]. While a significant amount of full length protein is still found in the ER fraction of the G213R membrane preparation, the 35 kDa fragment is observed in both lysosomal fractions, wt and mutant. Therefore, Ostm1 expression itself does not seem to be the limiting factor for the ER retained ClC-7 localization.

### The G213R ClC-7 mutant is still functional

The currents shown in Fig. 3 demonstrate that the wt as well as the G213R mutant in the absence and presence of Ostm1 are functional transporters. Since the currents in Fig. 3C are normalized to equal protein expression level, these data suggest that mutation as well as co-expression with Ostm1 modulate the activity of the individual transporter: the G213R mutation reduces the activity by ∼30% while co-expression with Ostm1 enhances the activity by a factor of ∼2. This is a relatively crude estimate, because the normalization procedure to the expression level using the EGFP fluorescence of the different preparations introduces a significant systematic error in the analysis. The error is due to cross contamination of the lysosomal and ER fractions (compare Fig. 2A - Calnexin) and the relatively imprecise determination of the protein concentration using the Bradford assay. However, the fact that the G213R mutant retains a similar relative activity of ∼70% of the wt in both cases, with and without Ostm1, supports the conclusion that G213R ClC-7 retains most of the transport activity of the wt.

Interesting is also the enhancement of the normalized transporter currents by Ostm1 (Fig. 3C). This is observed for both, the wt and the mutant. Because of the normalization to equal protein expression, it represents again a property of the individual transporter and is unrelated to expression density in the measured membranes. However, in view of the considerable errors involved in the normalization procedure it remains questionable whether this represents a true functional enhancement of the transporter function.

### Is ADO II caused by impaired transport activity of ClC-7 or by a lack of lysosomal ClC-7?

Mutations in ClC-7 or its β-subunit Ostm1 lead to severe pathological phenotypes [39]. In neurons, a disrupted lysosomal function leads to accumulation of lysosomal storage material and neurodegeneration [7]. Another example is the decreased acidification of the resorption lacuna in osteoclasts. This causes reduced resorption of mineralized bone as is the case in ADO II [18], [19].

Mutations can cause a lack of function by direct impairment of protein activity, by a lack of protein expression or by its incorrect trafficking. It has been suggested previously [19], that expression and localization of ClC-7 is normal in ADO II patients implying that this pathology is caused by a reduced functionality of G213R ClC-7. Our data seem to challenge this hypothesis although they are strictly valid only for rat ClC-7 and for the CHO expression system. On the other hand, rat ClC-7 is 96% identical to the human protein [8] and eukaryotic protein trafficking is believed to follow similar laws in different mammalian cell types.

As shown above, the transporter function of G213R ClC-7 is not abolished. At the same time localization studies demonstrate that G213R ClC-7 is retained in the ER while the wt is correctly targeted to the lysosomal membranes (Fig. 1A). Applied to ADO II this suggests that G213R related ADO II is predominantly caused by defective trafficking, while the impaired transporter activity of the mutant ClC-7 plays only a marginal role. Interestingly, osteoclasts from ADO II patients show a reduced total resorption pit area but the area of the individual pits was the same [18] implying that some of the ADO II osteoclasts are still fully competent of bone resorption while others are inactive. This agrees with the inhomogeneous cell population found in G213 ClC-7 CHO cells (Fig. 1C) where in some cells predominantly the correct lysosomal localization is found while in others the transporter is retained in the ER.

### SSM-based electrophysiology for drug discovery

Because of its involvement in bone pathology, ClC-7 is an interesting target for drug discovery. For example, inhibitors of ClC-7 are under discussion for the treatment of osteoporosis [22], [23], [25]. CHO cells were chosen because they produced only small background currents due to their low level of endogenous Cl^−^ conductance. Cl^−^ transporters like ClC-5 are not at all expressed in CHO [40] and also the tissue specific members of the ClC family like ClC-6 are not expected in CHO. ClC-3, which could be detected in western blot analysis (Fig. 2D) (possibly because of endosomal contamination of the membrane fractions) could together with endogenous ClC-7 be responsible for the small endogenous background currents in the CHO control preparations (Fig. 3A).

Assay quality is commonly estimated by the Z'- factor, with a Z'>0.5 being considered an “excellent” assay [41]. Our SSM-based electrophysiological assay for ClC-7 yielded a Z' = 0.78. To validate the assay a few plausible inhibitor candidates for ClC-7 were tested but no highly selective and high affinity compound was found that would be suitable as a drug candidate. Comparison of the determined inhibitor profile to that of ClC-3 and other chloride channels and transporters supports the assignment of the transient currents to ClC-7. ClC-3 is blocked by phloretin and tamoxifen, which was not the case in our experiments [42], [43]. Furthermore, our findings are in good agreement with osteoclast acidification and resorptions studies [44], where NPPB and the compound NS5818 [19], [45] could be identified as relatively potent inhibitors, while 9-AC, CPA and CPP had no effect on the osteoclast activity.

In conclusion, the inhibition study proves the suitability of SSM-based electrophysiology for screening of ClC-7 inhibitors. Among the small number of tested compounds no promising drug candidate for the inhibition of ClC-7 could be identified. For a realistic chance of detecting drug candidates larger compound libraries have to be screened using dedicated equipment which is now commercially available [20].
